# Health-care seeking behaviour among persons with diabetes in Uganda: an interview study

**DOI:** 10.1186/1472-698X-11-11

**Published:** 2011-09-26

**Authors:** Katarina Hjelm, Fortunate Atwine

**Affiliations:** 1 School of Health and Caring Sciences, Linnaeus University, Växjö, S-351 95 Växjö, Sweden; 2 Department of Nursing, Mbarara University of Science and Technology (MUST), Mbarara, Uganda

**Keywords:** Healthcare-seeking behaviour, Diabetes Mellitus, Complementary Alternative Medicine, Uganda

## Abstract

**Background:**

Healthcare-seeking behaviour in patients with diabetes mellitus (DM) has been investigated to a limited extent, and not in developing countries. Switches between different health sectors may interrupt glycaemic control, affecting health. The aim of the study was to explore healthcare-seeking behaviour, including use of complementary alternative medicine (CAM) and traditional healers, in Ugandans diagnosed with DM. Further, to study whether gender influenced healthcare-seeking behaviour.

**Methods:**

This is a descriptive study with a snowball sample from a community in Uganda. Semi-structured interviews were held with 16 women and 8 men, aged 25-70. Data were analysed by qualitative content analysis.

**Results:**

Healthcare was mainly sought among doctors and nurses in the professional sector because of severe symptoms related to DM and/or glycaemic control. Females more often focused on follow-up of DM and chronic pain in joints, while males described fewer problems. Among those who felt that healthcare had failed, most had turned to traditional healers in the folk sector for prescription of herbs or food supplements, more so in women than men. Males more often turned to private for-profit clinics while females more often used free governmental institutions.

**Conclusions:**

Healthcare was mainly sought from nurses and physicians in the professional sector and females used more free-of-charge governmental institutions. Perceived failure in health care to manage DM or related complications led many, particularly women, to seek alternative treatment from CAM practitioners in the folk sector. Living conditions, including healthcare organisation and gender, seemed to influence healthcare seeking, but further studies are needed.

## Background

The incidence of Type 2 diabetes mellitus (DM) is increasing worldwide, with a pandemic mostly affecting people in developing countries in Africa and Asia under development [1,2]. The pandemic is related to urbanization with longevity and changes of lifestyle, from a traditional active way of life to a modern sedentary style with unhealthy diets and obesity, combined with genetic susceptibility development [3] and probably also poverty [4].

Uganda is reported as having 560 000 registered diabetes patients and it is estimated that the number has passed a million, in a population of 28 million [5]. Figures are expected to double by 2025 [1,2]. Many are unaware of the disease and the healthcare system receives people at the hospitals with DM at very late stages - when they have unknowingly had the disease for years. Therefore, the number of people with diabetes registered in hospitals is not an indicator of the real disease burden in the community [1]. DM is a chronic, progressive disease with micro-and macro-vascular complications (affecting eye, kidney, lower extremity, heart) likely to develop over time in relation to glycaemic control [1]. Life expectancy is reduced because of increased morbidity and mortality [6]. Besides the impact on health, the economic costs of the disease and related complications are enormous, in healthcare and in loss of productivity [7,8].

DM needs to be treated by a holistic approach through dietary adjustment, exercise, medication (if needed), education and self-care besides regular follow-ups [3]. Nurses play a key role by acting as a co-ordinator in organising holistic care to meet patients needs based on individual beliefs about health and illness, aimed at teaching patients to become experts on their own disease and self-management.

Health services in Uganda are provided by the public and private sector [9]. The public health delivery system run by the government consists of the district health system (village health teams, health care centres II, III and IV (and district general hospitals) and regional and national referral hospitals. Services are free of charge with the exception of private wings in public hospitals. The private system includes private not for profit organisations (Catholic, Protestant, Orthodox and Muslim) and private health practitioners which charge user fees. Also the traditional and complimentary medicine practitioners (e.g traditional healers, herbalists, traditional bone setters, traditional birth attendants etc.) can be found but are not fully integrated in the health care system. There is a negotiation about the user charge, payment is relatively low and might be made in terms of available items (e.g chicken, goat etc.).

Diabetes care in Uganda is run within the public and private healthcare system. Some hospital diabetes clinics in outpatient care have been developed (World Diabetes Foundation 2008). The clinics are often based on physicians (GPs or internists) specialised in diabetology and non-specialised nurses. Health services in Uganda are provided by a mix of public, private for-profit and private not-for profit providers [10]. No national health insurance system exists. The government is the main provider of health services free for all users, but health services are underfunded and frequently drugs are unavailable which forces patients to purchase from private pharmacies or from the complimentary alternative medicine (CAM) sector. Despite increasing numbers of patients with DM, the healthcare sector and the government hospitals are not equipped to give appropriate healthcare because medical supplies are mostly insufficient and patients get prescriptions to go and buy elsewhere.

Limited information has been found about healthcare-seeking behaviour among patients with DM, and none focusing primarily on developing countries. The relation between treatment-seeking behaviour and compliance of diabetic patients in a rural area in South India showed that both government and private clinics were used by a large proportion of patients [11]. A switch between these two sectors might interrupt their glycaemic control and negatively affect health. Thus, it is important to explore where patients seek healthcare the first and second time, and the reason why they go for help.

A previous study of Ugandans diagnosed with DM showed that underlying living conditions such as affordability of drugs, food, equipment for self-monitoring of blood glucose, and different gender roles determined beliefs about health and illness and affected health-related behaviour including healthcare-seeking behaviour [12]. There was limited knowledge about DM and the management, and use of CAM, e.g. herbs, prayers etc. were indicated. A study of an Indian community in South Africa showed that CAM was mainly used for chronic conditions such as DM if healthcare failed to cure the underlying condition [13]. About half of the patients investigated did not inform their doctors about the use. Findings about the influence of educational level and socio-demographic characteristics such as age and gender on the use of CAM are contradictory in previous investigations [14,13]. The question is where healthcare is sought and from whom, and whether living in a strained economy affects healthcare-seeking behaviour and increases the use of CAM and other care providers outside the professional sector.

The aim of this study was to explore healthcare-seeking behaviour, including use of complementary alternative medicine and traditional healers in Ugandans diagnosed with DM. Further, to study whether gender influenced healthcare-seeking behaviour.

## Methods

### Design

In this study a qualitative descriptive study design was used. Data were collected by individual semi-structured interviews in order to give the participants the opportunity and freedom to express their individual views and thereby, reach a deeper understanding on the studied subject [15].

### Participants

A snowball sampling procedure was used, which started with that the interviewer (second author) asking somebody who knows a person diagnosed with DM for > 2 years. The respondent was then asked to identify another with the same condition. Participants were thus recruited from different places in the district in order to capture variation in healthcare-seeking behaviour. Inclusion criteria were: diagnosis of DM, duration of DM > 2 years, age > 20 years and without known psychiatric disorder. The sample comprised 24 persons diagnosed with DM, 16 women (aged 25-70, Md 53 years; see table 1) and 8 men (aged 35-67, Md 46 years) born and living in south-western Uganda. Most were treated with oral antidiabetic agents, married, had an educational level from secondary school or college, and had occupations of low socio-economic status. Thus, the demographic characteristics were similar to most people in Uganda [16], and they originated from an area of the country where 75% of the population is living and most belong to the Bantu speaking group. In Uganda there are about 40 different ethnic groups which are divided into four main groups based on their languages: Bantu speaking, West-and East Nilotic people, and people talking Sudanese languages. Two thirds of the population is Bantu speaking.

**Table 1:** Characteristics of the studied population.

Variable	Female N = 16	Male N = 8
Age (year)^1^	53 (25-70)	46 (35-67)
Duration of DM (year)^1^	5 (3-13)	6 (2-15)
Treatment (n)		
Diet	0	7
Oral agents	12	1
Insulin	4	0
Marital status (n)		
Married/cohabitant	14	6
Widow/er	2	0
Single	0	2
Educational level (n)		
Illiterate	1	1
Primary school	4	3
Secondary school	1	1
Tertiary level/college	8	3
University > 2 years	1	0
Socio-economic position (n)		
Low	10	5
Middle	5	3
High	1	0
Employment status (n)		
Gainfully employed	6	3
Retired	2	1
Unemployed	8	4
Self-reported complications related to diabetes (n)		
Eyes	10	5
Heart	8	5
Kidney	3	0
Feet/Lower extremity	7	4
Per vaginal itching	1	0
Impotence	0	2

^1 ^Median (range).

### Ethical considerations

The Faculty Research Ethical Committee (FREC) of Mbarara University of Science and Technology (MUST) approved the study. The investigation was carried out in accordance with the Helsinki Declaration and written informed consent was obtained from the respondents.

### Data collection

Data were collected in 2009. The interview started with structured questions on socio-demographic and medical data. Then a semi-structured interview guide with open-ended questions was used (Table 2). The main questions were: Where do you seek help if you have health problems? Why do you seek help? If healthcare fails, where do you seek help?

**Table 2:** Interview questions used.

Where do you seek help if you have health problems?
Why do you seek help?
If healthcare fails, where do you seek help?
Is help also searched from traditional healers?
If the answer is yes, from whom? For what reason?

The interview guide was based on findings and previous investigations [12,3,17] and peer-reviewed by nurses and general practitioners working in diabetes care. A pilot test was made with two men and two women (not included in the study), resulting in changes in the way questions were asked.

The interviews were held in a quite place without interaction of other people either in the respondent's home or workplace, and lasted for 45 minutes to one hour, were audiotaped and transcribed verbatim. Sixteen interviews were held in English (official language) and eight in the local language Runyankore, led by a bilingual female nurse (second author) not involved in management of the participants diabetes. Interviews in the local language were translated into English and then back-translated. The results might be limited to people with an educational level from secondary school or college and occupations classified as belonging to low socioeconomic status as language use may differ in different socio-economic groups. Runyankore is one of 40 indigenous languages in Uganda [16] belonging to the Bantu languages, which is spoken in most of the southern half of Uganda. The Bantu languages are mutually comprehensible.

### Data analysis

Collection and analysis of data proceeded simultaneously until no new information was added (saturation; [15]). Notes were made from the taped interviews as themes emerged. Respondents' statements were compared and patterns, contradictions and themes were searched for [18]. By reviewing each line of the texts, topics were identified and the material was condensed into content categories. The model for healthcare-seeking behaviour [19] was then introduced to give the main analytical categories [18] as previously described [12,3,17]. Healthcare can be sought from different sectors in the society [19]; the popular sector among family, friends or relatives, in the professional sector among health professionals, or from folk-healers in the folk sector specialising in different forms of healing, sacred or secular. Finally, the results from the analysis of men and women were compared to study the influence of gender.

To increase the trustworthiness of the findings, the content of the categories was checked by the first author [18,15]. A diabetes specialist nurse and a general nurse (first and second author) were thus involved in analysing the data and high agreement was found.

## Results

### Healthcare seeking

In all respondents, irrespective of gender, help with health problems was solely sought from the professional sector primarily, mainly from health care staff in public hospital or private for-profit clinics, and in some cases private for-profit nursing homes.

Secondarily predominantly health professionals in the professional sector was consulted for health problems, in females mainly from hospitals, health centres free of charge or private for-profit nursing homes, in contrast to males who mainly turned to private for-profit clinics or private for-profit nursing homes. In a few cases in both groups practitioners in the folk sector were consulted, to obtain herbs or certain fruit for treatment, or food supplement distributors in the folk sector.

I go to X main hospital...Then, I go to Dr [NAME] in the private nursing home. (F (= female) 7)

X hospital...private (for-profit) clinic (M (= male) 2)

I used herbs. Food nutrient distributors. I started DNLD products and then Tiansh (F 5).

When needing help with different health problems, the respondents, irrespective of gender, in all but two cases consulted health professionals, mainly doctors and nurses. The exceptions were persons who turned to friends in the popular sector:

Doctors and nurses in the clinic and the hospital (M3)

I get help from the doctors and nurses in the hospital. People have told me to use herbs like rukaka, omubirizi, omujuma, neem tree, avocado powder as tea. (F7)

### Reasons for seeking help

For the respondents, irrespective of gender, the main reason for seeking healthcare/help was symptoms related to DM or control of DM. Many described severe patterns of symptoms causing fainting or collapse. A few in each group had come to refill or buy medications.

Vaginal itching. Dizziness, dry tongue that I even fail to talk, severe thirst. (F 15)

General weakness, fall down and collapse, sometimes I find myself in the hospital, I don't know exactly what happens, I just fall and sleep. (F 8)

I go there to refill medicine when it is finished. I always get abdominal muscle pain, general weakness sometimes failing to carry myself, I also feel pins and needles in my feet. (M 4)

Females often sought help with reviews of the disease and for control of blood glucose. Many of the women described having 'joint pains', some had sought for symptoms related to infections such as 'cough', 'fever', and 'malaria', and a few had 'problems with breathing'. Also vaginal itching and high blood pressure were exemplified as reasons for seeking help. Among males, one told he had sought help with 'joint pains', another for 'abdominal muscle pain', and one for 'dysfunctional sex'.

### From whom was help sought?

Respondents, irrespective of gender, said that they had predominantly sought help in the professional sector from doctors or nurses in the hospital. Some had turned to private for-profit clinics. One man told he had sought help from a private pharmacy and another claimed he relied on self-care measures, e.g. drinking soft drinks to treat low blood glucose.

Doctors and nurses in the hospital (M1)

Private (for-profit) clinic I go to, it is owned by a midwife. I go there because it is near and I use it as first aid facility. (F8)

I always go to get other drugs or to write for me and buy medicine from the private pharmacy because drugs at the hospital they are always out of stock. (M5)

Unable to stand up...Dizziness and falling down. I know what to buy and usually carry sweets, water, or a bottle of soda. Pepsi Cola or Coca. (M3)

#### Healthcare seeking in case of failed healthcare

In cases of failed healthcare all males, except one, had sought help from the folk sector, to obtain local herbs or Chinese medicine from traditional healers or food supplements from food nutrient distributors. One man wished to use food supplements but didn't have money to buy them, and another had been advised to use bitter herbs (omubirizi ngaroitan) but feared complications and thus hesitated:

I was convinced to take Chinese medicine but stopped it when I developed high blood sugar because I stopped the medicine for diabetes. I took it for one month (M4)

People talk about food supplements like Tiansh but I can't buy them because I don't have money. (M5)

Among women, those who experienced failed healthcare most had also used help from the folk sector, such as local herbs from traditional healers or food supplements (e.g. Omega 3, zinc) from food nutrient distributors in the folk sector. In some cases only the professional sector (hospital or eye clinic at the hospital) or the professional sector in combination with family and friends in the popular sector were sought for help. In one case the folk sector and the popular sector were combined, as the person got food supplements from food nutrients distributors and local herbs from friends.

I have much pain in my legs so I go to Kazaire (herbalist) for extracted local medicine in bottles but it is very expensive. For eye problems I go to R eye clinic. (F 3)

I get help from my daughter-in-law who takes me to another private (for-profit) clinic, Dyapharm, that provides Chinese medicine. (F 1)

I go to the hospital and R eye clinic. Food nutrient distributors for Golden Neo-Life Diamitte (DNLD). I tried Omega 3 to cure diabetes (F4).

However, in both groups there were some who had not experienced failed healthcare.

### Healthcare seeking from traditional healers

The number of females who had also sought help from traditional healers was roughly the same as those who had not, and one stated she had done it before but stopped because of the expenses and because it had deteriorated her condition.

Yes. Extracted herbal medicine...Kazaire gives medicine in powder form. Aloe Vera powder, when I use it I get some relief. (F4)

No, I have not tried elsewhere. (F2)

I get local herbs. Severe urination. If it is not Thursday for the diabetes clinic I use herbs or if I don't have money to buy the tablets I take bitter herbs. (F 5)

Before coming to X hospital, I used herbal medicine...failed to raise money and the condition worsened. (F 15)

With a few exceptions males had not sought additional help from traditional healers. Most had used herbs to treat DM and lower blood glucose but also for pain relief or treatment of high blood pressure. The reasons stated for not seeking help from traditional healers in the folk sector were mainly that they didn't believe in it or only tested it. Others said it was 'expensive' and one person stated that the 'money instead was needed to buy life-saving medications'. Fear of changes in blood glucose control was also evident.

For me, I don't believe in going to visit traditional healers. From health education by our doctor in the hospital we were advised not to leave diabetes medicine. There is no cure for diabetes and if you stop diabetes drugs you will go into coma and die. So I fear going into coma (F 10).

About half of the females had sought help from traditional folk healers, mainly to treat their diabetes or hypertension and in a few cases for treatment of pain. One had used it before but had stopped because of expense and being weakened by the treatment.

Only two of the males stated seeking help from traditional healers in the folk sector; both received herbs, one to lower blood pressure, the other to cure the diabetic disease. However, two of the men had not sought help from traditional healers but had got advice from friends in the popular sector to go there and used herbal medications.

The reasons a couple of females stated for not seeking help from traditional healers were that they *'don't believe in it'*, while a few men and women *'feared the use because of the risk of worsening' *the diabetic disease and blood glucose control; one in each group could not afford it.

## Discussion

This study is unique in exploring healthcare-seeking behaviour in diabetic persons living in a developing country. The main results showed that healthcare was mainly sought among doctors and nurses in the professional healthcare sector and predominantly because of severe symptom patterns related to DM and/or glycaemic control. Females more often focused on follow-up of DM and problems with joint pain and infections, while males described fewer problems. Among those who thought healthcare had failed, most had turned to traditional healers in the folk sector for prescription of herbs or for food supplements, more women than men. Males more often turned to private for-profit clinics while females to a higher extent used governmental institutions free of charge.

The main strength of this study is the focus on the participants' own perspective, which provides insights on individuals' perspectives, experiences, and behaviour that might contribute to a better understanding of the subject studied [15]. However, different understandings of terms/concepts used in the interviews might have influenced the results. An example is 'traditional healers', which might have a broader meaning including use of local herbs, but also could have a limited focus on treatment by witchcraft with different rites. The influence was minimised by the interviewer elaborating on the questions and probing particularly for the use of local medicine and herbs. It was advantageous to use an interviewer from the same culture, speaking the same language as the respondents.

Recruitment of respondents was made as snowball sampling in the community instead of only from the diabetes clinic in order to get a broader representation and avoid bias from professional healthcare. A major question to pose is whether the results reflect true CAM use, as a previous investigation from South Africa [13] showed that a low percentage of CAM users felt it necessary to inform their doctors about the use. However, the interview climate here was open and the respondents willing to share their information, but underestimation cannot be ruled out. The chief limitation of qualitative studies stated is the inability to generalise data [18,15]. However, the results are transferable to populations/contexts similar in characteristics as data have been carefully collected and analysed, with the main aim of understanding instead of seeking explanations [18,15].

The predominance of women in the sample (n = 16 vs 8 men) might be seen as a limitation. However, Type 2 diabetes mellitus affects women in a higher extent than men [1].

The reason for seeking care from the folk sector and mainly using herbs and food supplements when perceiving healthcare as failed is in accordance with a previous investigation of CAM use in an Indian community in South Africa [13]. This illustrates that CAM is not used as a complement to conventional treatment [14], but instead an alternative, indicating limited economic resources, as shown in Ugandans with DM [12]. Availability of drugs at the hospital might also explain the behaviour. In Uganda health services are underfunded and drugs are frequently unavailable, which forces patients to purchase from others [10]. Herbal medicines are more readily available than diabetes medications. Another important factor is individual beliefs about health and illness, e.g. discussing the influence of natural and supernatural forces causing DM and use of traditional herbs and rites, which might influence behaviour [12]. It is important to notice that the diabetic patients are seeking help from the folk sector to be cured of DM and health problems that professional diabetes care is unable to manage, particularly chronic pain, indicating poor control of DM and development of complications [6]. This is in agreement with the main reasons for CAM use by Indians in South Africa [13]. The main reasons for not using CAM or turning to traditional healers were factors such as not believing in its effects, fear of worsened conditions and high expense. An independent association with CAM use has been shown in Malaysians with a higher educational level and higher income [14], and the pattern of care was better and of higher quality in health-insured persons [20], thus illustrating the influence of living conditions on CAM use and health-seeking behaviour. Living conditions are important not only for beliefs about health and illness [12] but also for health-seeking behaviour.

The results of this study, with healthcare mainly being sought from the professional sector and the folk sector in case of failed healthcare, contradict the pattern hypothesised in the theoretical frameworks described by Helman and Kleinman [21,19], stating use of folk healers in the first case and professional care in the last case in persons from developing countries. This is possibly explained by the fact that use of health services is the result of an interaction process between factors related to the individual, the healthcare system and the context in which it occurs [22]. Further, a limitation is the lack of empirical testing of the model described by Helman [21].

In this study many described severe symptoms related to DM or control of DM as a reason for seeking help, and CAM, which is an indication of poor glycaemic control. Thus, careful follow-up and diabetes self-care education is needed, not least to raise awareness of the severity of DM and to maintain health. Gender influenced health seeking behaviour, as males more often used private for-profit clinics where care need to be paid for, while females more often used free governmental care. Women also focused on regular follow-up of DM and reported more health problems. The findings indicate dissimilarities between males and females in access to economic resources, severity of disease and health awareness. In general, females have been shown to have higher health awareness and be more prone to health-related activities than males [23], also evident in DM [24,25]. A previous study of utilisation of private care in diabetic persons has shown a relationship with health status and social factors [26]. The decision to use private care was associated with worse health status. This is in contrast to our findings and might be related to differences in data collection, sample size, and health care systems, including funding of care; further studies are needed. It is important to recognise that women in Uganda generally do not, and are not expected to, control cash income or economic assets. They remain in the subsistence sector where their economic contribution is not valued [27]. In a society with male dominance, particularly in economic life, with continuing acceptance of the traditional authority of men [28] and a worsening economic crisis where women have to expand their income-generating activities, both men and women have to cope with new economic pressures [27] and women depend on free government hospitals as men control the money and thus have easier access to private health care. The risk of a switch between different care givers is then greater in women, entailing a risk of interrupting compliance and glycaemic control, negatively affecting health [11]. This might explain both the more severe disease pattern described and the healthcare-seeking behaviour.

The distribution of the diabetes epidemic is argued to be produced by poverty [4]. The cumulative effects of structural constraints on healthy lifestyles and lack of a right to adequate medical care are results of poverty, possibly affecting women to a higher degree than men. Systemic and structural conditions need to be considered in diabetes care [29].

## Conclusions

It is important to consider that living conditions, individual beliefs and gender-related issues determine healthcare-seeking behaviour. Nurses play a key role in assessing information about people's beliefs and behaviours [12] including use of CAM to meet individual needs/preferences of their clients, to prevent switching between different caregivers, thus threatening appropriate treatment [11]. In this work it is particularly important to pay attention to women as they live with strained economic resources often controlled by their husbands [27], contributing to the need to use care providers outside the professional health care sector.

Further, it is important to develop well organised diabetes care focused on diabetes education, raising awareness about DM and its severity and self-management to reach good glycaemic control to prevent development of costly complications [3]. Thus, the consumption and costs of drugs, for the individual and society, could be minimised.

In diabetes care it is important to consider that use of CAM is widespread in developing countries [14] and that the use of traditional healers has been described as a parallel health care system [30]. Co-operation with exchange of knowledge is important [31] for delivering high-quality diabetes care without delay. Treatment modalities for DM among traditional and faith healers should be noted by health workers when developing health education programmes [32], and need to be included in training of health care staff. Education about potential benefits in the light of limited available evidence of effectiveness of CAM is very important for developing an integrated model of health care provision [14].

The central role of the nurse in assessing data and planning care for persons with DM to promote health needs to be recognised and developed, not least in light of the rapidly growing pandemic of DM [3] predominantly affecting populations in developing countries in Africa and Asia [1].

In conclusion, Ugandans diagnosed with DM mainly sought help from nurses and physicians in the professional healthcare sector, females more often in free governmental institutions and males from private-for-profit clinics. In case of perceived failure of healthcare to manage DM or related complications many, particularly women, turned to traditional healers and herbalists in the folk sector, practising CAM for treatment with traditional herbs or food supplements. Otherwise CAM was not used as many did not believe in it, found it too expensive, or feared the risk of worsening their disease. It was thus indicated that living conditions, including the health care organisation, and gender issues influenced healthcare-seeking behaviour, but further studies are needed.

## Competing interests

The authors declare that they have no competing interests.

## Authors' contributions

Both authors were involved in the study design, data analysis, manuscript preparation and critical revisions. FA was also responsible for data collection, and KH provided supervision as well as technical and material support. Both authors have read and approved the final manuscript.

## Pre-publication history

The pre-publication history for this paper can be accessed here:

http://www.biomedcentral.com/1472-698X/11/11/prepub
